# How is childhood development of immunity to *Plasmodium falciparum *enhanced by certain antimalarial interventions?

**DOI:** 10.1186/1475-2875-6-161

**Published:** 2007-12-04

**Authors:** Colin J Sutherland, Christopher J Drakeley, David Schellenberg

**Affiliations:** 1 Department of Infectious and Tropical Diseases, London School of Hygiene and Tropical Medicine, Keppel Street, London, WC1E 7HT, UK

## Abstract

The development of acquired protective immunity to *Plasmodium falciparum *infection in young African children is considered in the context of three current strategies for malaria prevention: insecticide-impregnated bed nets or curtains, anti-sporozoite vaccines and intermittent preventive therapy. Evidence is presented that each of these measures may permit attenuated *P. falciparum *blood-stage infections, which do not cause clinical malaria but can act as an effective blood-stage "vaccine". It is proposed that the extended serum half-life, and rarely considered liver-stage prophylaxis provided by the anti-folate combination sulphadoxine-pyrimethamine frequently lead to such attenuated infections in high transmission areas, and thus contribute to the sustained protection from malaria observed among children receiving the combination as intermittent preventative therapy or for parasite clearance in vaccine trials.

## Background

A significant reduction in the intolerable global burden of malaria morbidity and mortality is unlikely without the large-scale implementation of effective and sustainable prevention. However, it has been recognized for more than a decade that measures which reduce the level of exposure may interfere with the natural acquisition of immunity to malaria [1]. Direct testing of this hypothesis has been hampered by the lack of validated immunological markers of protection.

The suggestion that any short-term protection afforded by insecticide-impregnated materials (IIM) may be followed by longer-term increased susceptibility, or "rebound", applies equally to other interventions that reduce or prevent infection. For this reason, clinical trials of malaria vaccines and of intermittent preventive therapy (IPT) have used extended post-intervention follow-up to detect any rebound of excess malaria among recipients of the intervention. Published controlled trials of preventative interventions including IIM, IPT and vaccination are considered, and findings discussed in the light of evidence that low-dose blood stage infections of *Plasmodium falciparum *elicit effective and sustained immunity against clinical malaria [2].

It is hypothesized that the antimalarial sulphadoxine-pyrimethamine (SP), because of its long serum half-life and its activity against developing hepatic parasite stages, generates low dose blood stage inocula and attenuated infections, and can thus act as an ideal blood-stage vaccine when used for malaria prevention in highly endemic areas.

## Intervention 1: Insecticide-treated curtains in Burkina Faso

An ambitious programme initiated in 1994 to provide a high-coverage of IIM in Oubritenga Province, Burkina Faso, was associated with a reduction in entomological inoculation rate of >90% [3]. All-cause mortality fell over six years by at least 19% with no evidence from long-term follow-up that mortality was shifted to older-aged children [4]. Chloroquine treatment failure rates did not differ between protected and unprotected intervention villages [5].

In the absence of immunological markers, the acquisition of immunity to malaria may be demonstrated by the ability to clear resistant parasites under treatment with a failing drug [6,7]. Diallo and colleagues [8] measured parasite clearance rates after chloroquine treatment among 409 children in Oubritenga infected with parasites of the *pfcrt-*76T chloroquine resistant genotype, comparing those under lifelong IIM protection, with those without IIMs in their village. IIM-protected children were significantly more likely to successfully clear resistant parasites after chloroquine treatment, compared to unprotected children (OR 1.80, 95% C.I. 1.15 – 2.80; P = 0.01). After stratification, children under three years of age remained significantly better at clearing resistant parasites than their unprotected peers, whereas children IIM-protected and unprotected at a later age were equally able to clear their infections. Thus, in the fourth and fifth years of life, unprotected children had levels of immunity similar to those enjoyed by children who have been protected by IIM.

These findings suggest that IIM-protected children in Burkina Faso commonly acquired protective immunity earlier. This may be because the protection afforded by IIM delays, on average, the age at which children are first challenged by *P. falciparum *infections. Older children are more likely to mount an effective immune response, preventing further progression of disease. The process of acquiring immunity to malaria in these children is likely to involve interplay between:

• the risk of infection with *P. falciparum *leading to a disease episode

• the age at which children first encounter infection.

## Intervention 2: Anti-sporozoite vaccines

The anti-sporozoite *P. falciparum *vaccine RTS, S is now under extensive testing in clinical trials in sub-Saharan Africa. Phase II controlled trials of the vaccine in Gambian men [9] and Mozambiquan children [10,11] demonstrated protective efficacy of 30 – 45% against *P. falciparum *parasitaemia, but differed in one remarkable respect. Vaccine-elicited parasitological benefit was transient in Gambian men, having diminished within 16 weeks of vaccination, whereas RTS, S-vaccinated children in Mozambique enjoyed a reduction in malaria episodes through 18 months of follow-up [11]. A parsimonious explanation for this discrepancy in the observed duration of protection afforded by RTS, S is that vaccinated children gained enhanced anti-parasite immunity, compared to controls, that the men already enjoyed irrespective of vaccination status. As RTS, S appears to offer protection to only a proportion of those vaccinated, it is also likely that incomplete protection in some vaccinated individuals impairs release of post-liver-stage merozoites without completely preventing it [12]. This may lead to attenuated blood-stage parasite infections, and support the development of blood-stage immunity [2]. Thus, by partially and transiently preventing maturation of liver-stage parasites, RTS, S may be eliciting effective anti-blood-stage immunity in a proportion of vaccinated children, but not in adults.

The development of protective blood-stage immunity by this proposed mecanism would be dependent on:

• the likelihood of natural parasite challenge during the transient period of anti-sporozoite protection

• the level of immunity at the time of vaccination.

## Intervention 3: Intermittent preventive treatment

In a placebo-controlled trial in 700 infants in Tanzania, Schellenberg *et al *[13,14] investigated IPT with SP, administered at 3, 6 and 9 months of life at the time of normal scheduled immunizations. This study demonstrated 59% efficacy (95% C.I. 41–72%) against clinical episodes of malaria up to 12 months of age. Extended follow-up to age 2 years, a period of 15 months after the last dose of IPT, demonstrated persistent protection of 36% (95% C.I. 11–53%) against clinical malaria. The authors conclude that, in this setting, IPT in infants could be facilitating the development of protective immunity in these children.

Under moderate to high transmission intensity, it can be imagined that a subset of IPT recipients are naturally challenged by *P. falciparum *infections during a period of waning blood levels of the component drugs of SP. This could readily lead to attenuated blood-stage infections that do not flourish, and thus do not cause clinical malaria, but persist at low density, providing a challenge capable of eliciting protective immunity. This explanation is consistent with the results of a placebo-controlled clinical trial of the *P. falciparum *blood-stage vaccine Combination B, in which 120 participants (aged between five and nine years) were randomized into placebo or vaccine arms, with or without pre-intervention clearance of parasitaemia by SP [15]. A crude, uncorrected comparison of parasite prevalence during 18 weeks of follow-up in the two placebo arms suggests that SP acts like a very good vaccine (OR 0.167; 95% C.I. 0.044 – 0.599; P = 0.003). This analysis is confounded by both a higher prevalence of pre-treatment parasitaemia in the placebo arm that did not receive SP (37% vs 17%), and by the several weeks of effective prophylaxis afforded by SP, but nevertheless is consistent with the hypothesis presented here: that SP can enhance the acquisition of immunity in children under intense infection pressure. This effect was not observed in a recent study in adult vaccinees and controls, some of whom were given a prophylactic dose of SP prior to surveillance [16]. Key parameters of SP-enhanced immunity appear to be:

• the probablility of infection during the period of effective drug levels

• the level of immunity before IPT/chemoprophylaxis is given

## Hypothesis

Having presented evidence supporting the notion that low dose *P. falciparum *infections enhance development of immunity, it is now proposed that presumptive administration of SP to young children in moderate to high transmission areas can accelerate their acquisition of protective immune responses. This occurs because, firstly, the elimination half-lives of sulphadoxine (11 days) and pyrimethamine (3 days) in serum provide extended pharmacological protection against the emergence of new blood-stage infections from the liver, but become permissive of such new infections as serum drug levels wane in due course. Secondly, SP provides a level of causal prophylaxis against liver-stage infection that is poorly understood [17-19]. Liver-stage malaria parasites are particularly dependent on folate synthesis as a single sporozoite nucleus must initiate a staggering 14 mitotic divisions over 7–14 days to release >10^4 ^progeny merozoites into the blood. It is thus likely hepatic stages are exquisitely sensitive to anti-folate drugs such as SP, but no detailed studies have been published.

In particular, it is unknown whether resistance-associated mutations in the *pfdhfr *and *pfdhps *loci also protect hepatic parasites against SP, and it is a plausible possibility that SP remains effective against so called "resistant" parasites during rapid hepatic stage DNA replication, prior to further expansion of the total parasite burden during blood-stage infection. This offers an explanation for the continued utility of SP in preventative applications even where clinical cases commonly fail under SP treatment.

## Testing the hypothesis

Low-dose blood-stage infections enhance the acquisition of immunity against clinical malaria, perhaps partly by thwarting the immune suppression effects of clinical disease [20]. Sustained benefit is profoundly dependent on the likelihood of natural challenge during the period of protection (and thus on transmission intensity), on previous parasite exposure and on the level of immunity of the individual. Testing this hypothesis will require careful analysis of the impact of drug suppression on the acquisition of immunity in children with and without prior malaria infections.
